# Quantitative syntheses of permethylated *closo*-1,10-R_2_C_2_B_8_Me_8_ (R = H, Me) carboranes. Egg-shaped hydrocarbons on the Frontier between inorganic and organic chemistry†

**DOI:** 10.1039/c8ra06640j

**Published:** 2018-11-14

**Authors:** Mario Bakardjiev, Oleg L. Tok, Aleš Růžička, Zdeňka Růžičková, Josef Holub, Drahomír Hnyk, Jindřich Fanfrlík, Bohumil Štíbr

**Affiliations:** Institute of Inorganic Chemistry, Academy of Sciences of the Czech Republic Husinec-Řež 1001 Czech Republic stibrb@seznam.cz; Department of General and Inorganic Chemistry, Faculty of Chemical Technology, University of Pardubice Studentská 573 532 10 Pardubice Czech Republic; Institute of Organic Chemistry and Biochemistry of the Czech Academy of Sciences Flemingovo nám. 2, 166 10 Prague 6 Czech Republic

## Abstract

Electrophilic methylation of the *closo*-1,10-R_2_C_2_B_8_H_8_ (1) (R = H or Me) dicarbaboranes at higher temperatures or thermal rearrangement of the 1,6-R_2_C_2_B_8_Me_8_ (3) compounds at 400–500 °C generated the B-permethylated derivatives *closo*-1,10-R_2_C_2_B_8_Me_8_ (2) in quantitative (>95%) yields. The compounds exhibit extreme air stability as a consequence of a rigid, egg shaped hydrocarbon structures incorporating inner 1,10-C_2_B_8_ carborane core.

## Abstract 2

Reported are procedures leading to highly stable egg-shaped hydrocarbons with inner *closo*-1,10-C_2_B_8_ carborane core inside the methyl sheath.

## Graphical abstract

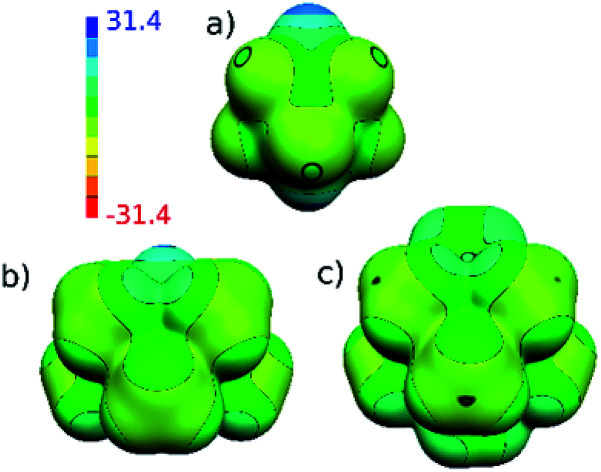

## Introduction

Methods for cage substitution on the cage of *closo*-1,10-C_2_B_8_H_10_ (1a) generally parallel those employed for the larger C_2_B_10_H_12_ icosahedral carboranes.^1^ The CH hydrogens in 1a are sufficiently protonic in character to undergo lithiation with butyllithium in ethereal solvents, generating the mono- and dilithio derivatives.^2^ The C-lithiated species afford the main entry to alkyl, aryl, carboxyl, silyl, and other C-substituted derivatives *via* treatment with appropriate reagents. For example, exo-polyhedral metal complexes,^3,4^ and the silyl-linked mixed-carboranes^5^ have also been prepared by this route along with numerous C-metallated compounds containing main-group metals.^1^ The only B-substitution processes so far reported are, however, the direct reaction between 1a and Cl_2_ affording the B-perchloro species 1,10-H_2_C_2_B_8_Cl_8_ (ref. 2) and those leading to a series of halo derivatives 1,10-H_2_C_2_B_8_H_7_-2-X.^6^ The successful B-methylation experiments in the 12-vertex carborane series,^7–9^ together with those achieved by our group in the B-methylation of *closo*-1,6-C_2_B_8_H_10_,^10^*arachno*-6,9-C_2_B_8_H_14_,^11^ prompted us to extend boron-methylation strategy to the most stable members of the 10-vertex *closo* series, *closo*-1,10-R_2_C_2_B_8_H_10_ (1) (where R = H or Me), which have been now relatively easily available.^12^ In this article we would like to present electrophilic reactions with methylation agents leading to quantitative permethylation of B-vertexes under the formation of rigid, hydrocarbon–boron structures of egg shape that exhibit outstandingly high stability.

## Results and discussion

**Scheme 1:**
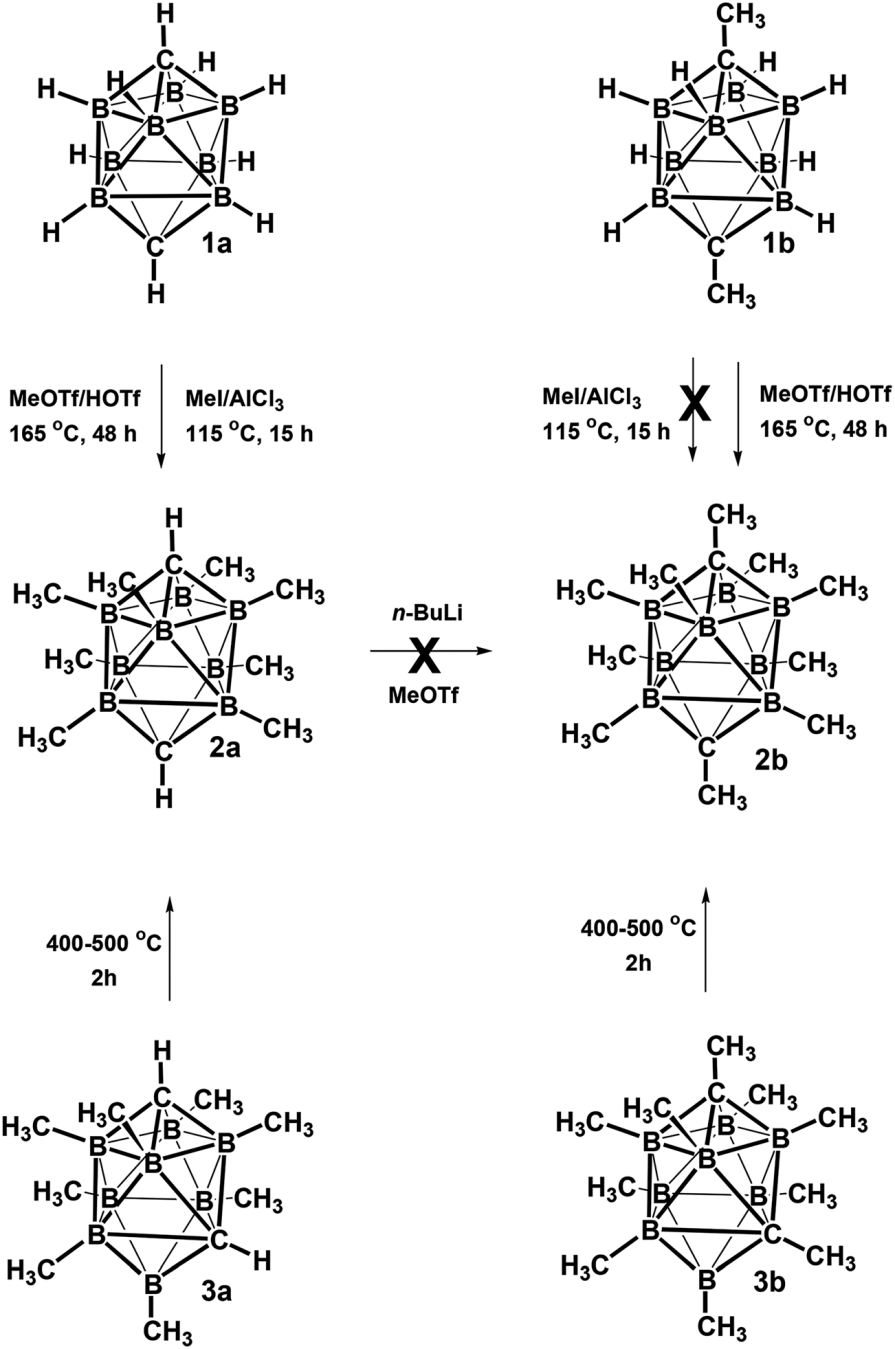
Quantitative syntheses of permethylated derivatives of the *closo*-1,10-R_2_C_2_B_8_H_8_ family.

The electrophilic CH_3_I/AlCl_3_ methylation of carborane *closo*-1,10-H_2_C_2_B_8_H_8_ (1a) (Scheme 1) led on heating at 115 °C for 15 h to exclusive formation of the B-permethylated dicarbaborane *closo*-1,10-H_2_C_2_B_8_Me_8_ (2a) in practically quantitative yield (>95%). The scheme also shows that the CH_3_OTf/HOTf (Tf = SO_2_CH_3_) methylation proceeded excellently at 165 °C for 48 h, giving again a quantitative yield of 2a (>95%). It is, however, interesting that the CH_3_I/AlCl_3_ methylation (115 °C, 15 h) of *closo*-1,10-Me_2_C_2_B_8_H_8_ (1b) has completely failed, while the CH_3_OTf/HOTf methylation of 1b proceeded smoothly at 175 °C for 48 h, giving again a quantitative yield of the decamethylated *closo*-1,10-Me_2_C_2_B_8_Me_8_ (2b). This finding is in accord with that observed in the comparable 12-vertex *closo*-1,12-Me_2_C_2_B_10_Me_10_ series (reflux, 20 h, 91% yield).^9^ As also observed for the latter species, an attempt at methylating the CH1,10 vertexes in 2a*via* the Li^2+^ salt as in Scheme 1 has failed, too. The difficulty in forcing this reaction to completion must be due to the relative lack of reactivity of the CH vertices present in 2a, which is exacerbated by the steric protection afforded to each CH vertex by the methyl groups of the four surrounding BMe, vertices.

**Scheme 2:**
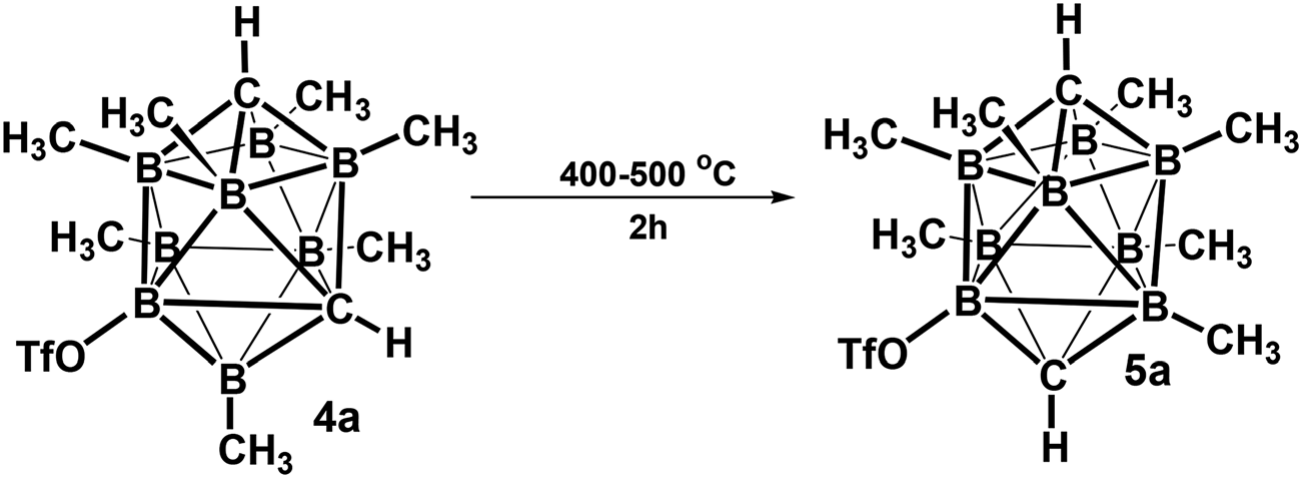
Quantitative synthesis of the permethylated derivative *closo*-1,10-R_2_C_2_B_8_Me_7_-2-OTf.

Another straightforward route leading to quantitative formation of permethylated dicarbaboranes 2 consists in thermal isomerisation of *closo*-1,6-R_2_C_2_B_8_Me_8_ (3) carboranes (R = H 3a or Me 3b)^10^ by heating at 400–500 °C in a sealed tube for 2 h. A similar thermal isomerisation principle could be also applied to the substituted derivatives, for example to *closo*-1,6-H_2_C_2_B_8_Me_7_-8-OTf (4a),^10^ which underwent cage rearrangement to afford 97% of the *closo*-1,10-H_2_C_2_B_8_Me_7_-2-OTf (5a) upon a similar heating, as shown in Scheme 2.

**Fig. 1:**
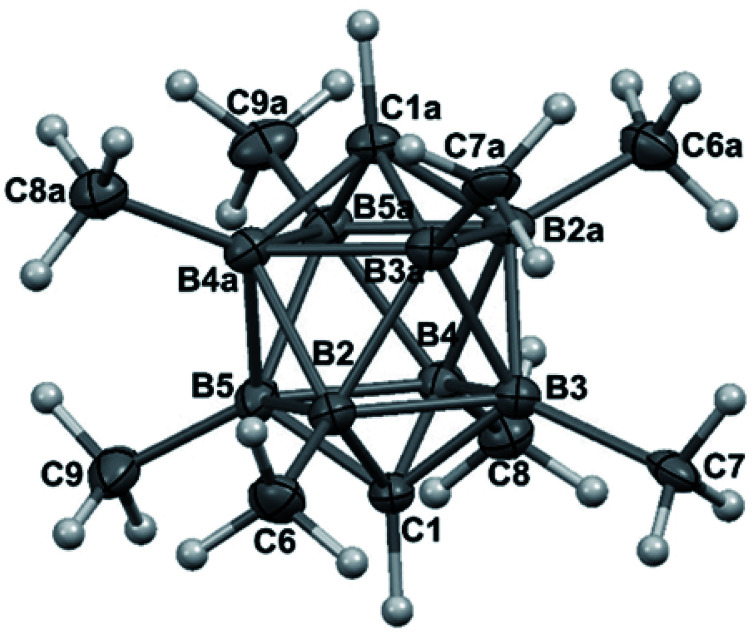
ORTEP (30% probability level) representation of the molecular structure of *closo*-1,10-H_2_C_2_B_8_Me_8_ (2a). Selected bond lengths (Å): C1–B2 1.600(4), C1–B4 1.595(4), B3–B2 1.870(4), B2–B3a 1.821(4), mean B–CH_3_ 1.639(4); angles (°): B5–B2–B3 89.99(18), C1–B2–B3a 107.2(2), B2–B3a-B3 61.70(17), B5–C1–B2 71.39(17).

**Fig. 2:**
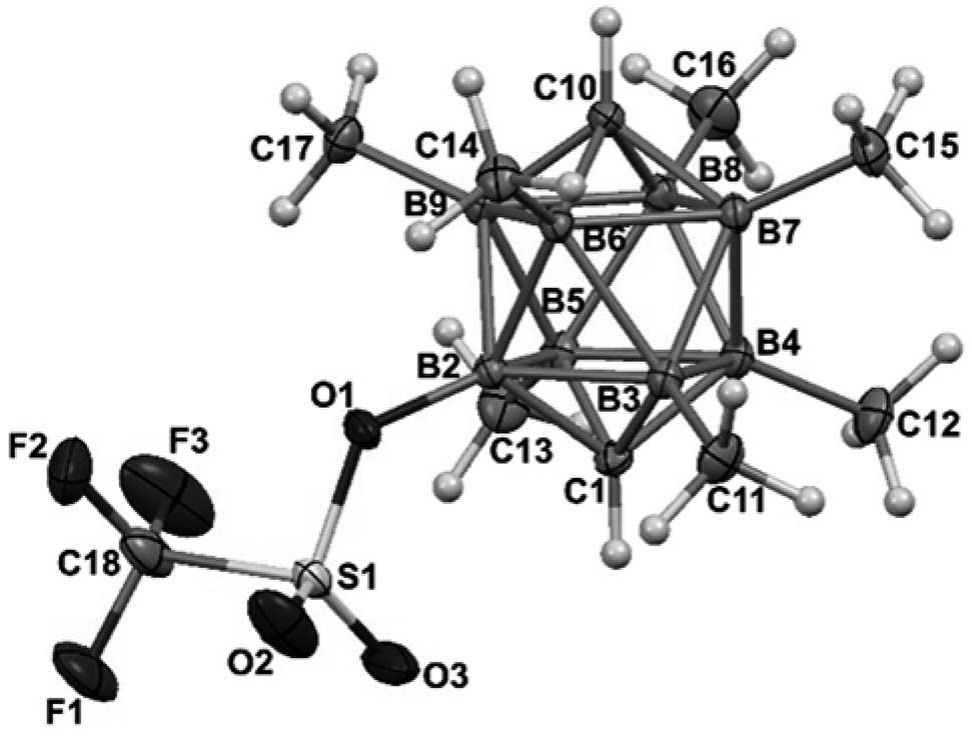
ORTEP (30% probability level) representation of the molecular structure of *closo*-1,10-H_2_C_2_B_8_Me_7_-2-OTf (5a). Selected bond lengths (Å): C1–B2 1.584(2), C1–B4 1.598(3), B2–B3 1.851(3), B2–B6 1.806(3), mean B–CH_3_ 1.578(3), B2–O 1.453(2), mean C10–B 1.601(3), angles (°): B5–B2–B3 91.96(11), C1–B2–B6 109.72(13), B2–B6–B3 61.03(10), B2–C1–B5 70.64(12).

**Fig. 3:**
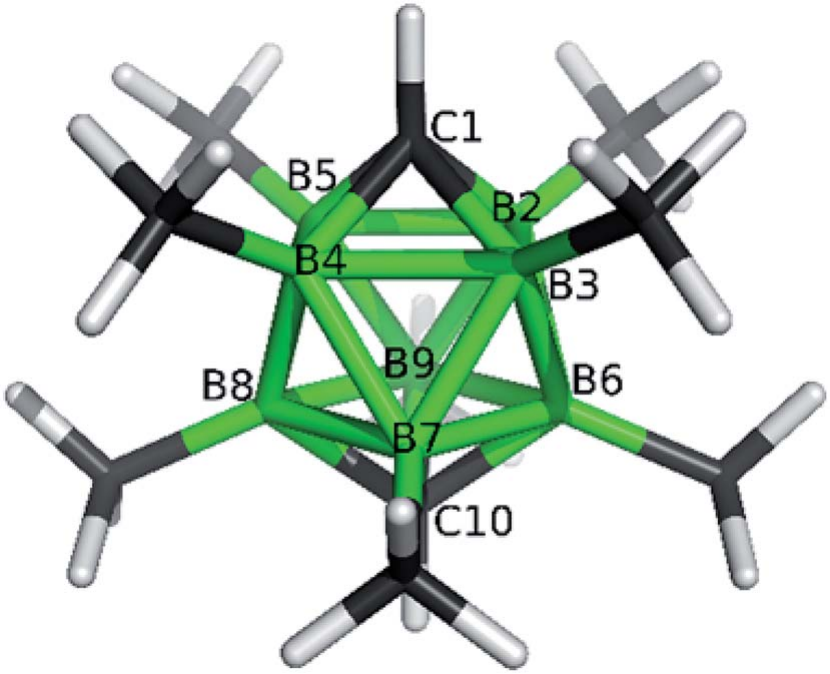
Molecular model for *closo*-1,10-H_2_C_2_B_8_Me_8_ (2a) as obtained from the MP2/TZVP optimization. The selected internal coordinates are (distances in Å, angles in °): C1–B2 1.602, C1⋯C10 3.329, B2–B3 1.866, B2–B6 1.818, B6–B7 1.866.

**Fig. 4:**
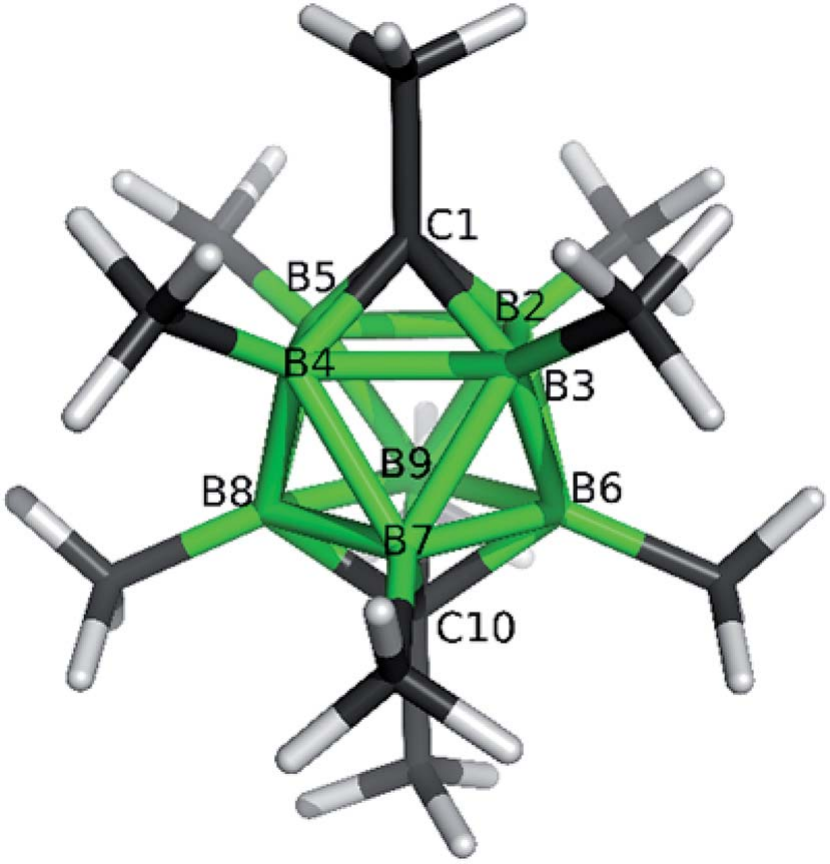
Molecular model for *closo*-1,10-Me_2_C_2_B_8_Me_8_ (2b) as obtained from the MP2/TZVP optimization. The selected internal coordinates are (distances in Å, angles in °): C1–B2 1.607, C1⋯C10 3.367, B2–B3 1.856, B2–B6 1.812, B6–B7 1.858.

The structures of derivatives 2a and 5a were determined by an X-ray diffraction study (see Fig. 1 and 2). Both carboranes adopt the expected bicapped Archimedean antiprismatic geometry with two unsubstituted apical CH1,10 vertexes along with eight or seven BMe groups, respectively. The structure 5a unambiguously confirms the B2-substitution with the O-SO_2_-CF_3_ group and permethylation in all other B-positions of the cluster. Unfortunately, the crystals of the permethylated 2b were not found suitable for crystallographic studies and the 2a/2b pair was therefore geometry optimized at the MP2/TZVP level (Fig. 3 and 4).

The optimization revealed that the comparable B–B, C–B, and B–Me bonding vectors are very similar to those found crystallographically for 2a and 5a. The computation has also led to a good agreement between theoretical and experimental *δ*(^11^B) values for 2a and 2b (max. deviation less than 3 ppm), for individual values see ESI.†

**Fig. 5:**
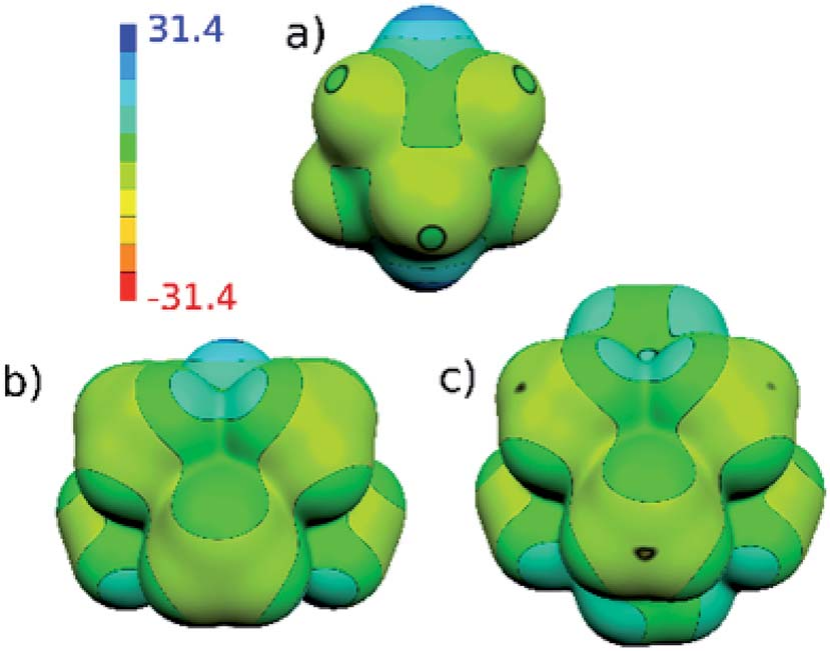
Computed (HF/cc-pVTZ) electrostatic potential (ESP) surface for (a) 1a, (b) 2a and (c) 2b. The color range of the ESP in kcal mol^−1^.

The HF/cc-pVTZ calculations of the electrostatic potential (ESP) surface show that the parent 1a has hydridic hydrogen atoms, which can form dihydrogen bonds,^13^ the hydridic B-bound hydrogens in 2a and 2b are now replaced by methyl groups of amphiphilic character.^14^ The hydrogen atoms of the methyl groups have positive ESP surface and the exo-skeletal carbon atoms have negative ESP surface (see Fig. 5). From the viewpoint of electron transmission, Me groups behave as weak electron acceptors, when compared to toluene, xylene, hexamethylbenzene *etc.* This is in accord with the 1,6-isomers of the same molecular shape^10^ and the same also applies to CMe methyl groups; the lower electron density at C1,10 in 2b in relation to 2a (see C1⋯C10 body diagonals) may perhaps prove this concept. The electron transmission thus follows the established electronegativity concept (C in CH_3_ is more electronegative than C (cage)) reflecting the fact that exo-skeletal substituents are bound *via* classical 2-centre 2-electron bonds.^15^ This agrees with the concept elaborated by Viñas *et al.* on a hexamethylated *closo*-H_2_C_2_B_10_H_4_Me_6_ system.^16^ Conceivably, the whole ^11^B NMR spectra of 2a and 2b are significantly paramagnetically deshielded to high frequencies (*i.e.* downfield shifted) with respect to those of parent compounds, for the computed ^11^B NMR spectra see ESI.†

**Fig. 6:**
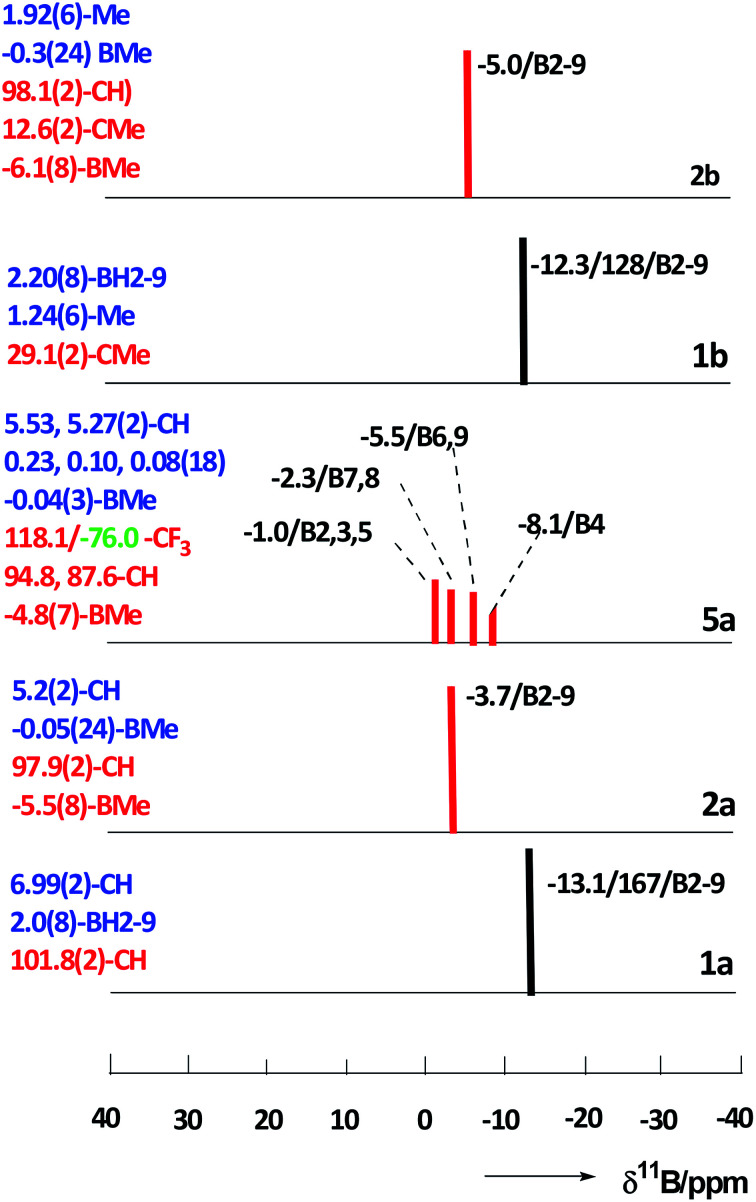
Stick diagrams comparing the ^11^B NMR chemical shifts and relative intensities for *closo* compounds 1,10-H_2_C_2_B_8_H_8_ (1a), 1,10-H_2_C_2_B_8_Me_8_ (2a), 1,10-H_2_C_2_B_8_Me_7_-2-OTf (5a), 1,10-Me_2_C_2_B_8_H_8_ (1b), and 1,10-Me_2_C_2_B_8_Me_8_ (2b) (red sticks = singlets, black = doublets). The data for individual cluster positions are ordered as: *δ*(^11^B)/^1^*J*_BH_ (where applicable)/assignment. ^1^H (blue text) and [^13^C,^19^F] (red and green text) NMR resonances (*δ* in ppm/TMS) are summarized on the left side of each diagram with relative intensities other than 1 in parentheses.

The constitution of all compounds isolated in this study is also in agreement with the results of multinuclear ^11^B, [^11^B–^11^B]-COSY,^17^^1^H, and ^13^C NMR measurements that led to complete assignments of individual cage BMe, BX, CH, and CMe units (for hardcopies of the NMR measurements, see ESI, Fig. S1–S13†). The multinuclear (^11^B, ^1^H and ^13^C) NMR spectra for all compounds isolated in this work are compared and depicted in Fig. 6, a general feature of the persubstituted compounds being the identity of the ^11^B and ^11^B–{^1^H}-decoupled NMR spectra that exhibit only singlet resonances.

**Fig. 7:**
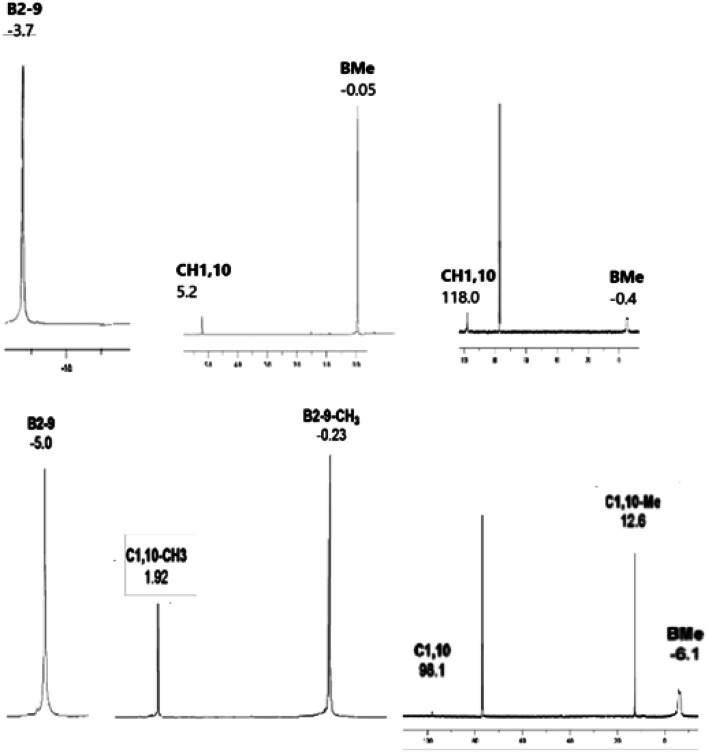
192.6 MHz ^11^B, 600 MHz ^1^H, and 150.9 MHz ^13^C NMR spectra of *closo*-1,10-H_2_C_2_B_8_Me_8_ (2a) (top) and *closo*-1,10-Me_2_C_2_B_8_Me_8_ (2b) (bottom).

The ^11^B NMR spectra of the *D*_4h_-symmetry derivatives 2a and 2b (Fig. 3, S7 and S10†), exhibit only one singlet due to equivalency of all B-positions; the corresponding ^1^H spectra (Fig. 6, S8 and S10†) consist of two sharp 1 : 12 or 1 : 4 singlets for 2a and 2b, respectively, attributed to CH1,10 (or Me1,10) and BMe resonances. The ^13^C–{^1^H} NMR spectra of carboranes of structure 2 (Fig. 6, S9 and S11†) show one lower-field C1,10 singlet along with a very broad (*J*_C–B_ coupling) high-field BMe resonance of relative areas 1 : 4, whereby that of 2b shows an additional C–Me signal. For NMR spectra of the key permethylated derivatives 2a and 2b, see Fig. 7.

On the other hand, the ^11^B NMR spectrum of the *C*_s_-symmetry compound 5a (Fig. 3 and S12†) displays 3 : 2 : 2 : 1 patterns of singlets with one coincidental overlap. The ^1^H spectrum of 5a (Fig. 6 and S13†) shows two different CH signals and four well resolved 2 : 2 : 2 : 1 patterns of BMe resonances in the high-field; the ^13^C–{^1^H} NMR spectrum contains two different low-field CH resonances together with a typical low-field CF_3_ quartet, apart from a broad, high-field BMe signal of intensity 7 (see Fig. S13†).

The ^19^F NMR spectrum of 5a shows one singlet resonance at −76.0 ppm, as expected.

## Experimental

### Materials and methods

All the reactions were carried out under argon atmosphere. Dichloromethane and hexane were dried over CaH_2_ and freshly distilled before use. Other conventional chemicals were of reagent or analytical grade and were used as purchased. NMR spectroscopy was performed at 400 and 600 Mz (for ^1^H), inclusive of standard [^11^B–^11^B]-COSY^17^ experiments (all theoretical cross-peaks were observed) leading to complete assignments of all resonances to individual cage B-vertexes. Chemical shifts are given in ppm to high-frequency (low field) of Ξ = 32.083971 MHz (nominally F_3_B·OEt_2_ in CDCl_3_) for ^11^B (quoted ± 0.5 ppm), Ξ = 25.144 MHz for ^13^C (quoted ± 0.5 ppm), and Ξ = 100 MHz for ^1^H (quoted ± 0.05 ppm), Ξ is defined as in ref. 18 and the solvent resonances were used as internal secondary standards. The starting carboranes of structures 1 and 3 were prepared according to the reported methods.^10,12^

### Dimethylation of *closo*-1,10-C_2_B_8_H_10_ (1a) on carbon vertices

A solution of 1a (120 mg, 1 mmol) in dry Et_2_O (*ca.* 10–20 ml) was cooled to −78 °C and then treated dropwise with 2.5 M LiBu (solution in hexane) (1 ml, 2.5 mmol) under stirring. The off-white slurry of the Li^+^ salt was stirred for additional 1 h prior to addition of methyl triflate (MeOTf, m.w. 164.1) drop by drop, (410 mg, 2.5 mmol) under cooling down in an dry-ice bath. The mixture was then left stirring for additional 2 h at room temperature. After adding 5 % hydrochloric acid (10 ml) under repeated cooling and shaking, the Et_2_O layer was separated and evaporated to provide a crude product *closo*-1,10-Me_2_C_2_B_8_H_8_ (1b) in practically a quantitative yield, as assessed by NMR spectroscopy (see Fig. S4–S6†).

### *closo*-1,10-R_2_C_2_B_8_Me_8_ (2) (where R = H 2a or Me 2b)

#### (a) Methylation with MeOTf

A solution of carboranes 1a or 1b (reaction scale ∼ 1.5 mmol) in neat MeOTf (5 ml) was treated with three drops of HOTf and the mixture was heated for 48 hours at 165 °C in a thick-walled reaction vessel equipped with a Teflon screw cap. The volatiles were evaporated and the residue extracted with hexane, the extract was filtered through a plug of silica gel, evaporated, and then sublimed at 150–180 °C (bath) to isolate white crystals of carboranes 2a and 2b in practically quantitative yields (97–98%). For NMR spectra, see Fig. 6 and S7–S12.†

#### (b) Methylation with MeI

A solution of carboranes 1a or 1b (reaction scale ∼ 1.0 mmol) in neat MeI (10 ml) was treated with anhydrous AlCl_3_ (*ca.* 14 mg, 0.14 mmol) and heated at 115 °C (oil bath) for 15 hours in a thick-walled thick-walled reaction vessel equipped with a Teflon screw cap. The volatiles were evaporated the residue subjected to extraction with pentane, filtered through a plug of silica gel, and vacuum sublimed as sub (a) to isolate 2a (yield 97%). Compound 2b has not been formed at all and 1b was recovered from the pentane extract in ∼80% yield.

#### (c) Thermal rearrangement of *closo*-1,6-R_2_C_2_B_8_Me_8_ (3) compounds (where R = H or Me)

Compounds 3a or 3b (reaction scale ∼ 0.5 mmol) were heated in a sealed tube at 400–500 °C (heating gun) for 2 hours. Sublimation as in preceding experiments led to essentially quantitative isolation of carboranes 2a and 2b. For 2a: MS (ESI^−^): *m*/*z* (max.) calcd 232.28, found 232.25; for C_10_H_26_B_8_ (m.w. 232.80) calcd 51.59 %C, 11.26 %H; found 51.21 %C, 11.15 %H. For 2b MS (ESI^−^): *m*/*z* (max.) calcd 260.31, found 260.31; for C_12_H_30_B_8_ (m.w. 260.85) calcd 55.25 %C, 11.59 %H; found 54.80 %C, 11.19 %H. For NMR spectra, see Fig. 6 and S7–S12.†

### Thermal rearrangement of *closo*-1,6-R_2_C_2_B_8_Me_7_-8-OTf (4a)

Compound 4a (74 mg, 0.2 mmol) was heated in a sealed ampoule at 400–500 °C (heating gun) for 2 hours. The resulting product was identified as *closo*-1,10-R_2_C_2_B_8_Me_7_-2-OTf (5a) and isolated on sublimation (bath temperature ∼ 150 °C) in essentially quantitative yield. For 5a MS (ESI^−^): *m*/*z* (max.) calcd 366.20, found 366.19; for C_10_H_23_B_8_O_3_SF_3_ (m.w. 366.84) calcd 32.74 %C, 6.32 %H; found 32.28 %C, 6.15 %H. For NMR spectra, see Fig. 6, S13 and S14.†

### Computational details

Magnetic shielding was calculated using the GIAO-MP2 method incorporated into Gaussian09 (ref. 19) utilizing the IGLO-II basis with the MP2/TZVP geometry and frozen core electrons. Electrostatic potentials were computed at the HF/cc-pVDZ level using Gaussian09 and Molekel4.3 (ref. 20) programs. It has recently been shown that this basis set size is sufficient for these purposes.^21^

### X-ray crystallography

The X-ray data for the derivatives 2a and 5a (colourless crystals by slow evaporation of a hexane solution) were collected at 150(2) K with a Bruker D8-Venture diffractometer equipped with Mo (Mo/K_α_ radiation; *λ* = 0.71073 Å) microfocus X-ray (IμS) source, photon CMOS detector and Oxford Cryosystems cooling device was used for data collection. The frames were integrated with the Bruker SAINT software package using a narrow-frame algorithm. Data were corrected for absorption effects using the Multi-Scan method (SADABS). Obtained data were treated by XT-version 2014/5 and SHELXL-2014/7 software implemented in APEX3 v2016.5-0 (Bruker AXS) system.^22^ Hydrogen atoms were mostly localized on a difference Fourier map, however to ensure uniformity of treatment of crystal, all hydrogen were recalculated into idealized positions (riding model) and assigned temperature factors *H*_iso_(H) = 1.2*U*_eq_ (pivot atom) or of 1.5*U*_eq_ (methyl). H atoms in methyl groups were placed with C–H distances of 0.96 while the hydrogen atoms of the C–H in the carborane cage were assigned according to the maxima on the difference Fourier map. *R*_int_ = ∑∣*F*_o_^2^ − *F*_o,mean_^2^∣/∑*F*_o_^2^, *S* = [∑(*w*(*F*_o_^2^ − *F*_c_^2^)^2^)/(*N*_diffrs_ – *N*_params_)]^1/2^ for all data, *R*(*F*) = ∑∣∣*F*_o_∣ − ∣*F*_c_∣∣/∑∣*F*_o_∣for observed data, w*R*(*F*^2^) = [∑(*w*(*F*_o_^2^ − *F*_c_^2^)^2^)/(∑*w*(*F*_o_^2^)^2^)]^1/2^ for all data. Crystallographic data for structural analysis have been deposited with the Cambridge Crystallographic Data Centre, CCDC no. 1573271 and 1573272 for 2a and 5a, respectively. Crystallographic data for 2a: C_10_H_26_B_8_, *M* = 232.79, orthorhombic, *Pbcn a* = 8.5140(13), *b* = 15.098(3), *c* = 12.670(2) Å, *β* = 90°, *Z* = 4, *V* = 1628.6(5) Å^3^, *D*_c_ = 0.949 g cm^−3^, *μ* = 0.045 mm^−1^, *T*_min_/*T*_max_ = 0.6416/0.7456; −10 ≤ *h* ≤ 11, −19 ≤ *k* ≤ 19, −15 ≤ *l* ≤ 16; 14 313 reflections measured (*θ*_max_ = 27.55°), 1875 independent (*R*_int_ = 0.0566), 136 with *I* > 2*σ*(*I*), 91 parameters, *S* = 1.078, *R*_1_(obs. data) = 0.0977, w*R*_2_(all data) = 0.2866; max., min. residual electron density = 0.792, −0.319 eÅ^−3^. Crystallographic data for 5a: C_10_H_23_B_8_F_3_O_3_S, *M* = 366.82, monoclinic, *P*2_1_/*c*, *a* = 13.0608(9), *b* = 9.8980(6), *c* = 16.0303(9) Å, *β* = 111.202(2) °, *Z* = 4, V = 1932.1(2) Å^3^, *D*_c_ = 1.261 g cm^−3^, *μ* = 0.200 mm^−1^, *T*_min_/*T*_max_ = 0.6787/0.7456; −16 ≤ *h* ≤ 16, −12 ≤ *k* ≤ 12, −20 ≤ *l* ≤ 19; 47 313 reflections measured (*θ*_max_ = 27.54°), 4445 independent (*R*_int_ = 0.0300), 3971 with *I* > 2*σ*(*I*), 241 parameters, *S* = 1.049, *R*_1_(obs. data) = 0.0502, w*R*_2_(all data) = 0.1431; max., min. residual electron density = 0.729, −0.628 eÅ^−3^.

## Conclusions

There are not too many reactions in the area of carborane chemistry that proceed quantitatively.^1^ To these rare cases belong syntheses leading to permethylated derivatives of *closo*-1,10-R_2_C_2_B_8_H_8_ (1) reported this work. It was shown that all B-positions in structures 1 can be furnished with methyl substituents, *via* electrophilic reactions with MeOTf or MeI reagents. In quantitative yields proceed also the 1,6- → 1,10-carbon rearrangement reactions of the isomeric compounds *closo*-1,6-R_2_C_2_B_8_Me_8_ (3). Moreover, the permethylated compounds, such as 2a and 2b, can be, in fact, envisaged as egg (or ellipsoid) shaped hydrocarbons (see Fig. 5) of remarkable air stability due to the protective sheath of the surrounding methyl groups. For example, the persubstituted 2a can be stored in air for at least a month without any noticeable change, while the unprotected 1a is decomposed in air within a couple of hours, especially in a solution. The less stability of the unprotected intermediate-sized carborane 1a derives from its non-icosahedral constitution (though it exhibits features of 3D aromaticity^23^). The quantitative yields and relative easiness of the synthesis predestinate these persubstituted derivatives for using in designed syntheses in specific areas of carborane chemistry as multipurpose reagents, for example in cluster-insertion/expansion or cage-degradation processes. Apart from this, such compounds are expected to exhibit extreme hydrophobicity, which can be made use of in various directions of chemical or biochemical research. Relevant experiments aimed at extension of permethylation chemistry are therefore in progress in our laboratories.

## Conflicts of interest

There are no conflicts to declare.

## Supplementary Material

RA-008-C8RA06640J-s001

RA-008-C8RA06640J-s002
