# Supplementary material for: Quantitative syntheses of permethylated closo-1,10-R2C2B8Me8 (R = H, Me) carboranes. Egg-shaped hydrocarbons on the Frontier between inorganic and organic chemistry
Source: RSC Adv. 2018 Nov 14;8(67):38238–44. doi: 10.1039/c8ra06640j (PMC9090134; doi:10.1039/c8ra06640j)
Supplement: RA-008-C8RA06640J-s001 [file RA-008-C8RA06640J-s001.pdf]

Electronic Supplementary Material (ESI) for RSC Advances.
This journal is © The Royal Society of Chemistry 2018

Supplementary Information

Quantitative Syntheses of Permethylated *closo*-1,10-$R_2C_2B_8Me_8$ (R = H, Me) Carboranes. Egg-shaped Hydrocarbons on the Frontier between Inorganic and Organic Chemistry

Mario Bakardjiev, Aleš Růžička, Zdeňka Růžičková, Josef Holub, Oleg L. Tok Drahomír Hnyk, Jindřich Fanfrlík, and Bohumil Štíbr,*

RSC Advan.

Selected NMR measurements ($CDCl_3$)

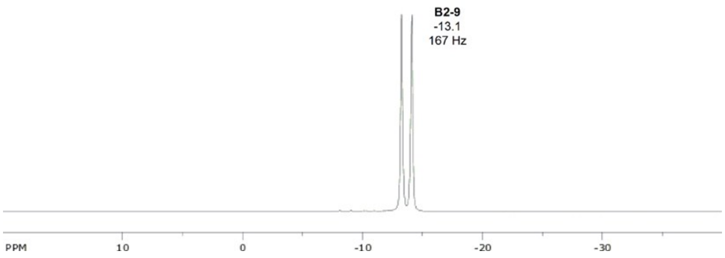

Figure S.1 190.2 MHz ^{11}B NMR spectrum of the starting *closo*-1,10-$H_2C_2B_8H_8$ (**1a**). Ordered as assignment/δ(^{11}B in ppm relative to BF_3OEt_2/ $^1J_{BH}$ in Hz.

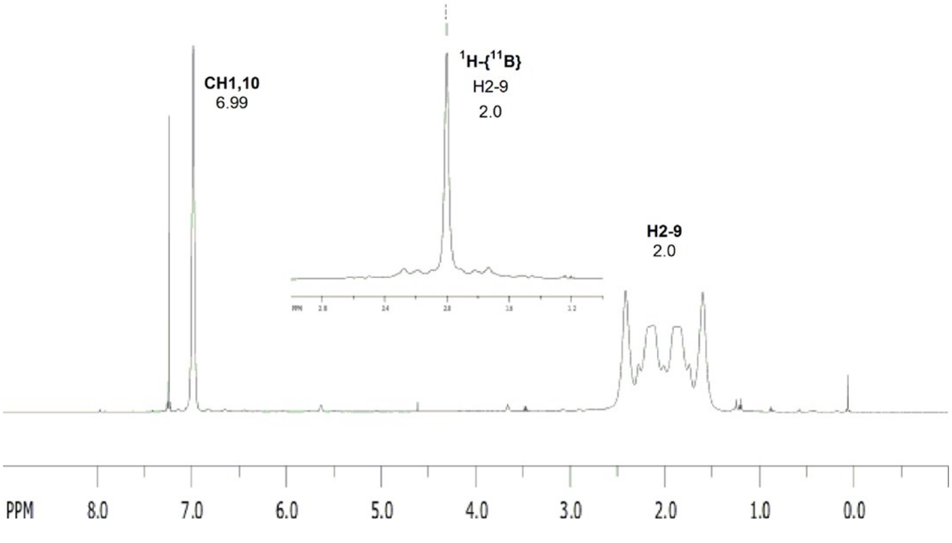

Figure S2. 600 MHz ^1H NMR spectrum of the starting *closo*-1,10-$H_2C_2B_8H_8$ (**1a**). Ordered as assignment/δ(^1H in ppm, relative to TMS). The quartet at 2.0 ppm reflects the $^1J_{BH}$ coupling.

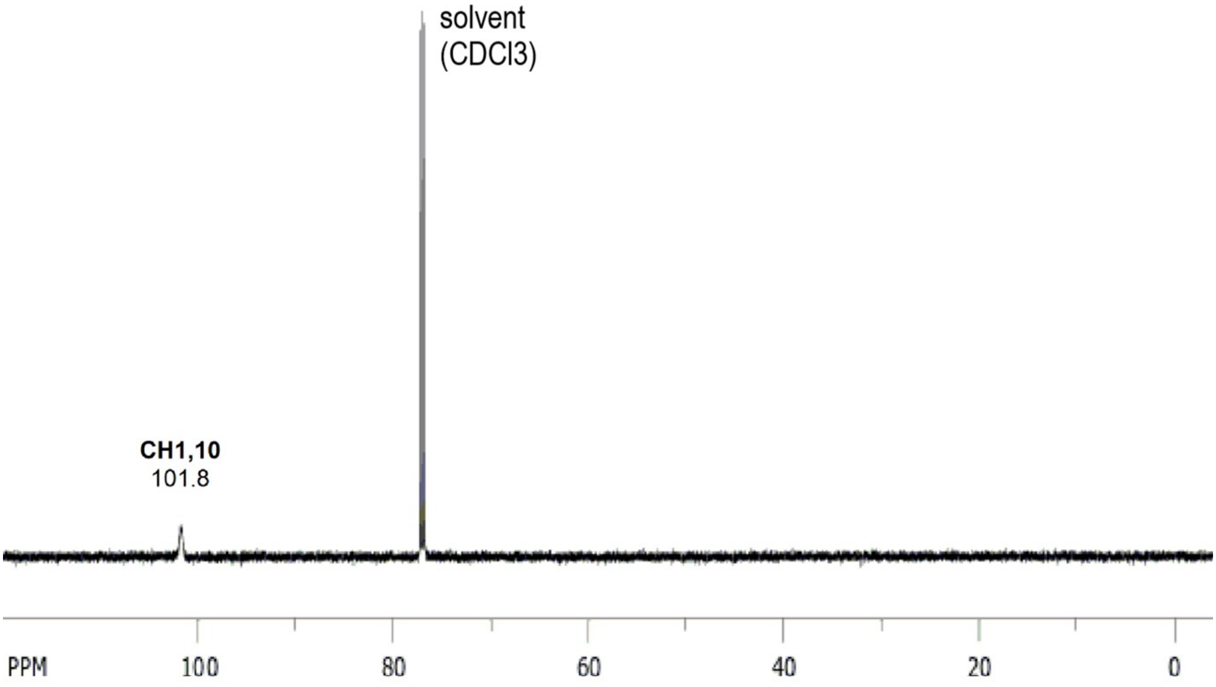

Figure S3. 150.9 MHz ^{13}C-{^{1}H} NMR spectrum of the starting *closo*-1,10-$H_2C_2B_8H_8$ (**1a**). Ordered as assignment/δ(^{13}C in ppm, relative to TMS).

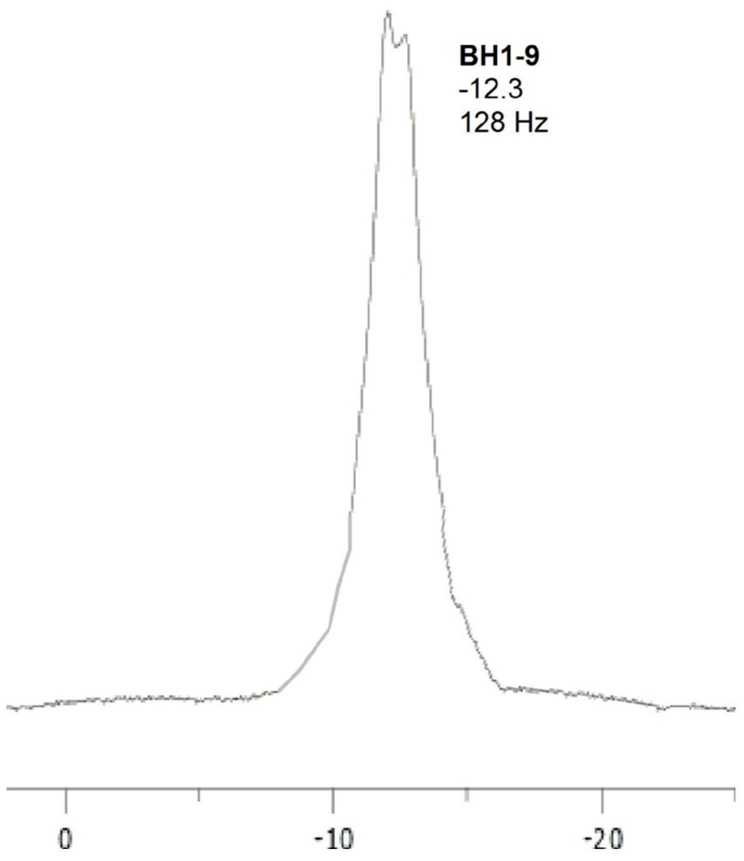

Figure S4. 190.2 MHz ^{11}B NMR spectrum of the starting *closo*-1,10-$Me_2C_2B_8H_8$ (**1b**). Ordered as assignment/δ(^{11}B in ppm relative to BF_3OEt_2/ $^1J_{BH}$ in Hz. The BH doublet is less pronounced, probably because of the effect of the CMe groups.

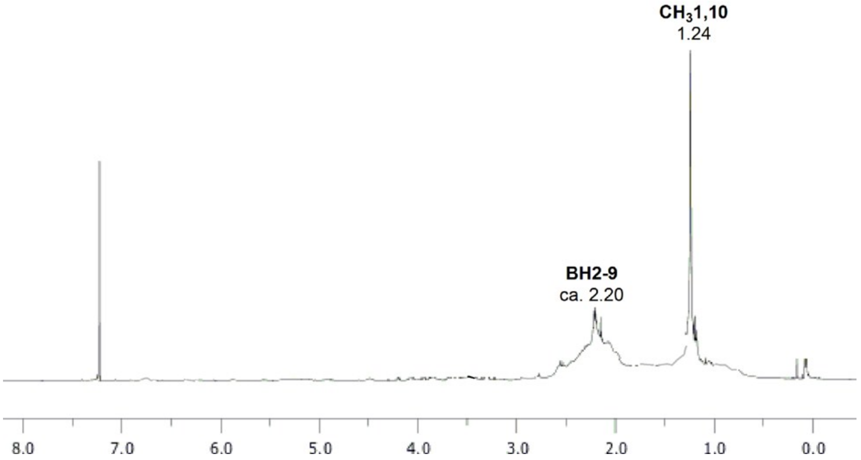

Figure S5. 600 MHz ^{1}H NMR spectrum of the starting *closo*-1,10-$Me_2C_2B_8H_8$ (**1b**). Ordered as assignment/δ(^{1}H in ppm, relative to TMS). The broader signal at ~2.20 ppm reflects the $^1J_{BH}$ coupling combined with long-range couplings due to the two non equivalent CMe groups. The high-field impurities near ~0.00 ppm are due to traces of silicon grease.

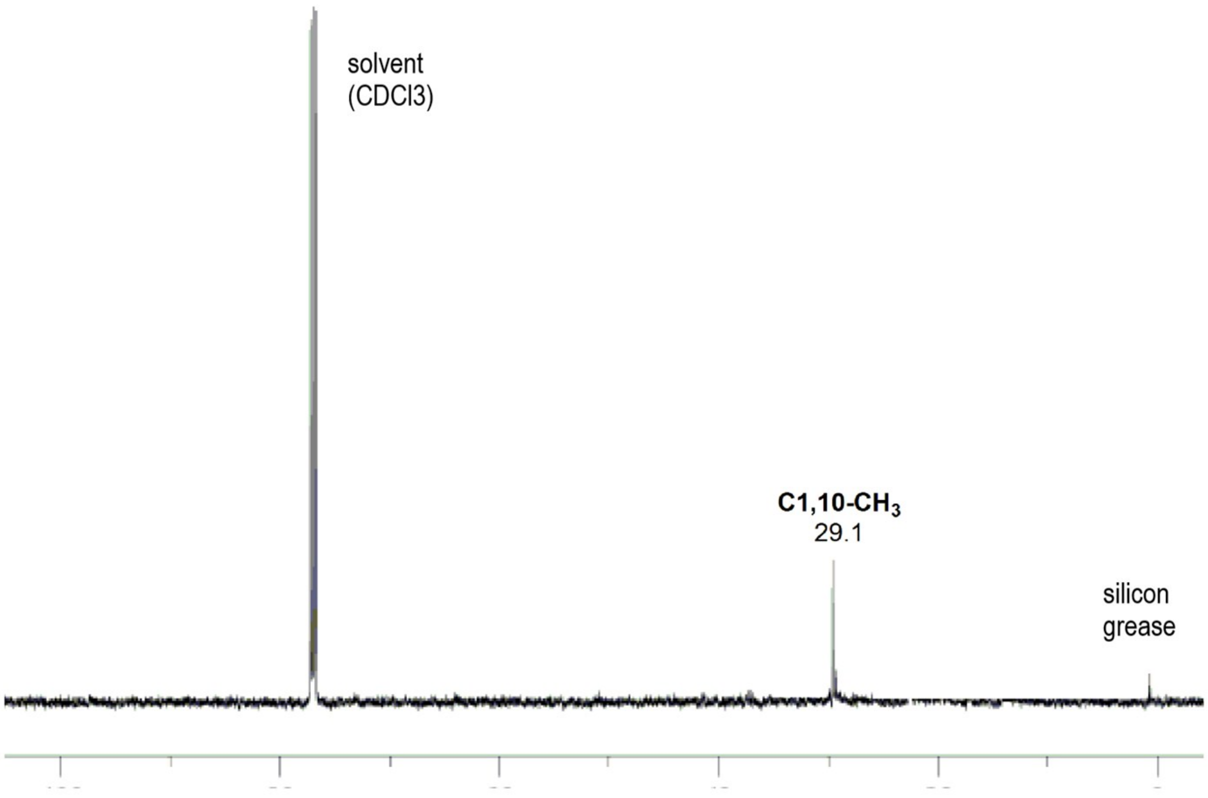

Figure S6. 150.9 MHz ^{13}C-{^{1}H} NMR spectrum of the starting *closo*-1,10-$Me_2C_2B_8H_8$ (**1b**). Ordered as assignment/δ(^{13}C in ppm, relative to TMS).

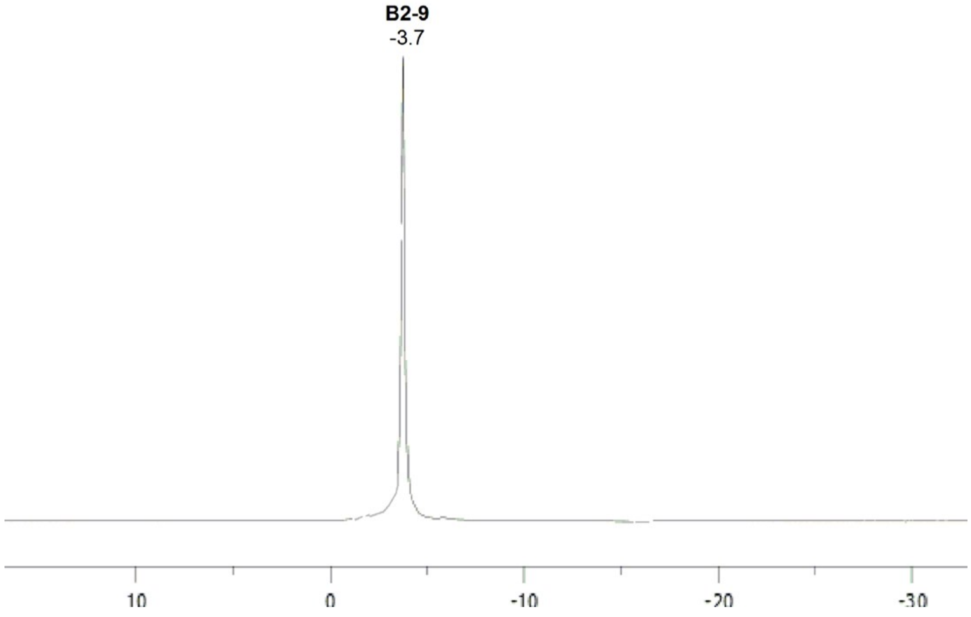

Figure S7. 190.2 MHz ^{11}B NMR spectrum of *closo*-1,10-$H_2C_2B_8Me_8$ (**2a**). Ordered as assignment/δ(^{11}B in ppm relative to BF_3OEt_2/ $^1J_{BH}$ in Hz. The singlet shape proves the persubstitution by Me groups in all B-positions.

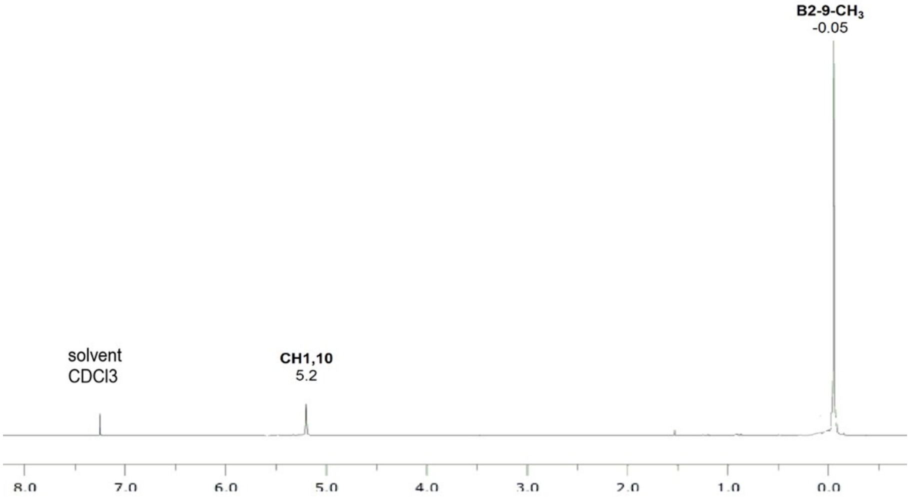

Figure S8. 600 MHz ^1H NMR spectrum of *closo*-1,10-$H_2C_2B_8Me_8$ (**2a**). Ordered as assignment/δ(^1H in ppm, relative to TMS). The high-field BMe signal at ~-0.05 ppm reflects absolutely clean permethylation in all B-sites.

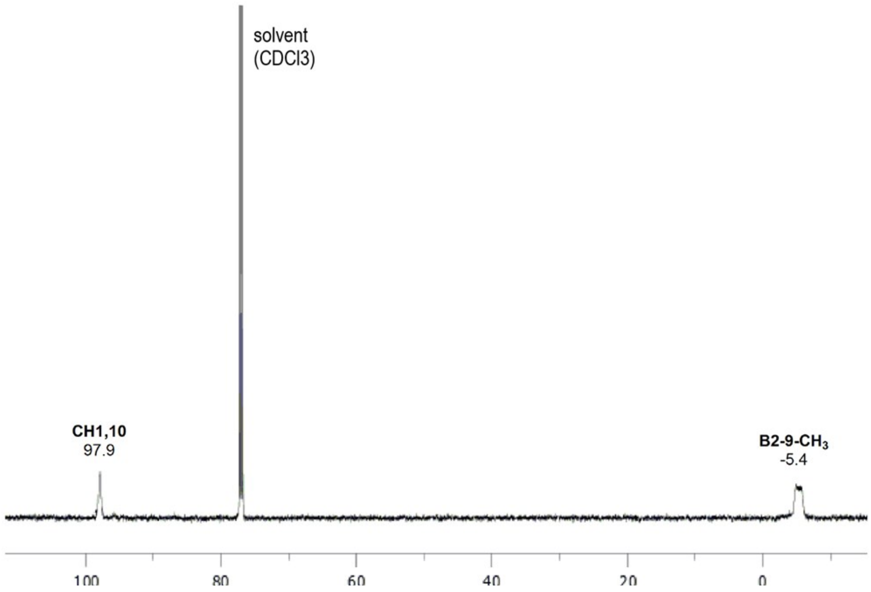

Figure S9. 150.9 MHz ^{13}C NMR spectrum of *closo*-1,10-$H_2C_2B_8Me_8$ (**2a**). Ordered as assignment/δ(^{13}C in ppm, relative to TMS).

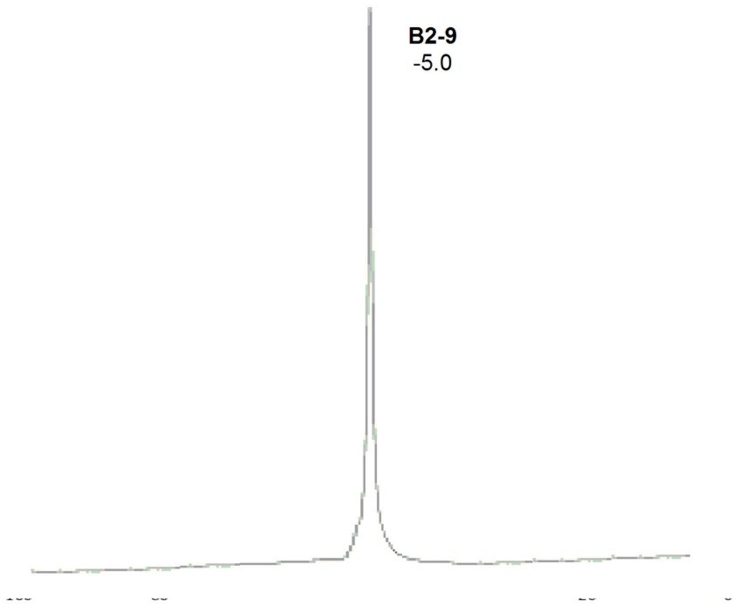

Figure S.10 190.2 MHz ^{11}B NMR spectrum of *closo*-1,10-$Me_2C_2B_8Me_8$ (**2b**). Ordered as assignment/δ(^{11}B in ppm relative to BF_3OEt_2). The singlet shape proves the permethylation in all positions.

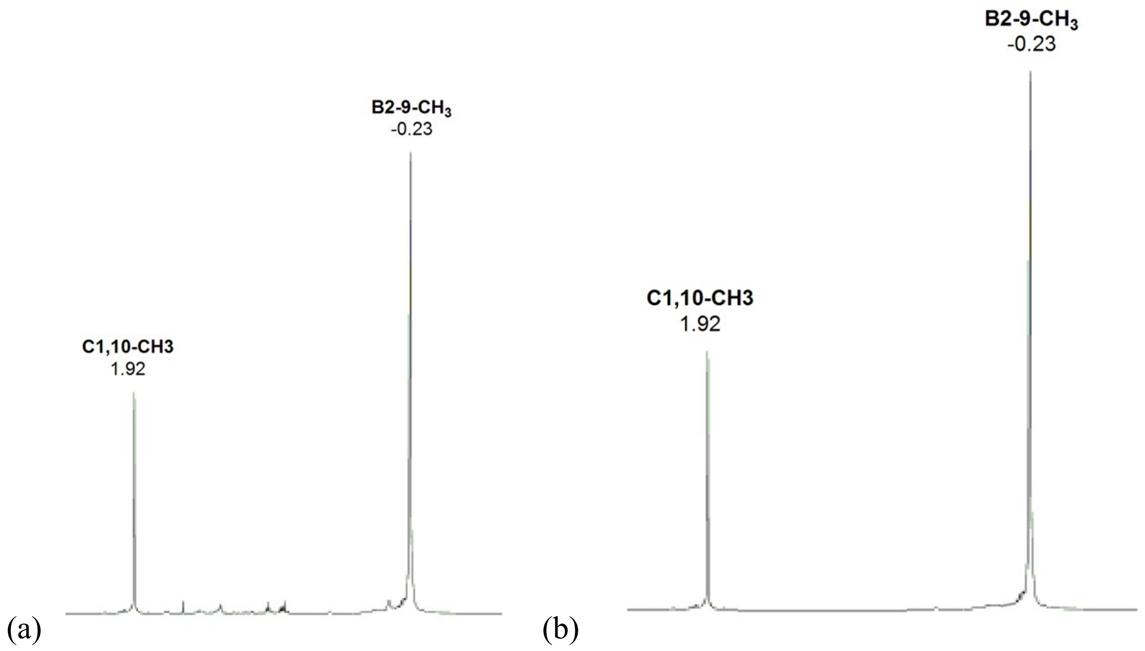

Figure S 11. 600 MHz ^{1}H NMR spectra of *closo*-1,10-$Me_2C_2B_8Me_8$ (**2b**) (a) crude product (b) after sublimation removing the minor organic impurities. Ordered as assignment/δ(^{1}H in ppm, relative to TMS).

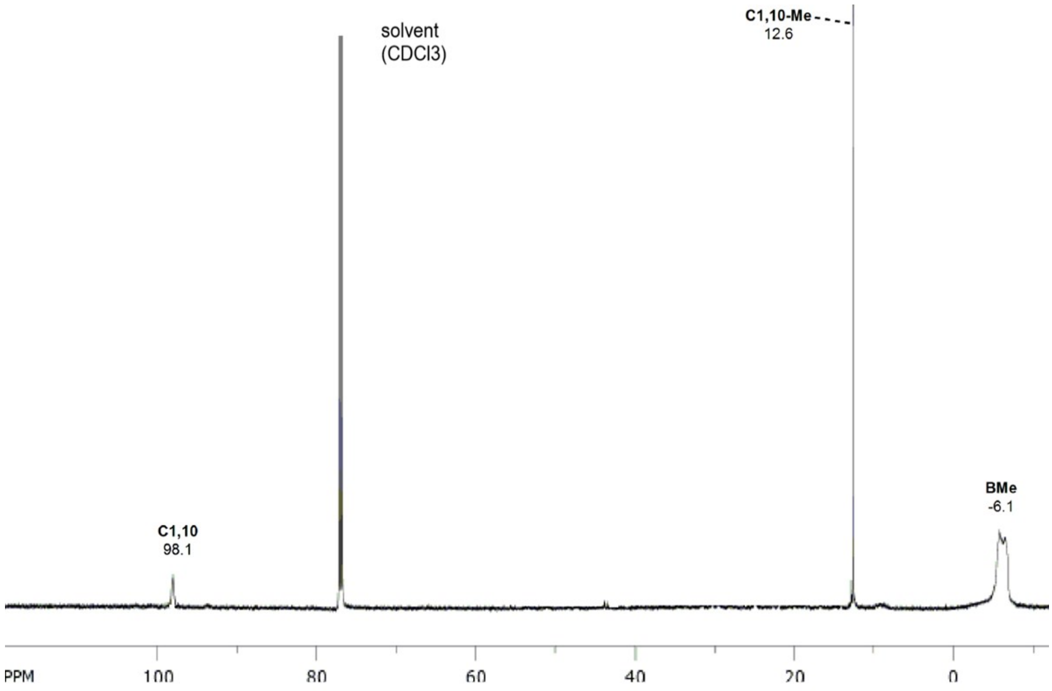

Figure S12. 150.9 MHz ^{13}C-{^{1}H} NMR spectrum of *closo*-1,10-$Me_2C_2B_8Me_8$ (**2b**). Ordered as assignment/δ(^{13}C in ppm, relative to TMS).

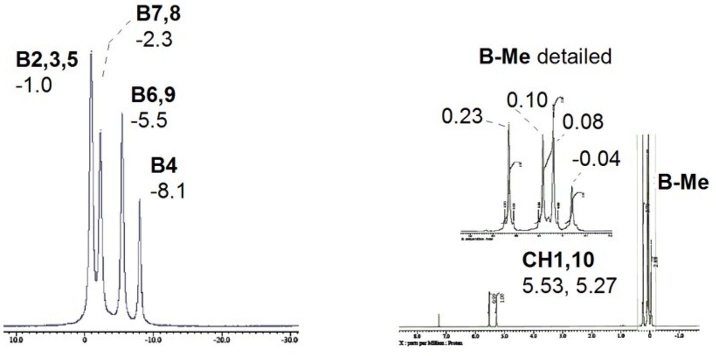

Figure S13. 190.2 MHz ^{11}B (left) and 600 MHz ^{1}H (right) NMR spectra of *closo*-1,10-$H_2C_2B_8Me_7$-2-OTf (**5a**). Ordered as assignment /δ(^{11}B in ppm relative to BF_3OEt_2) or assignment/δ(^{1}H in ppm, relative to TMS).

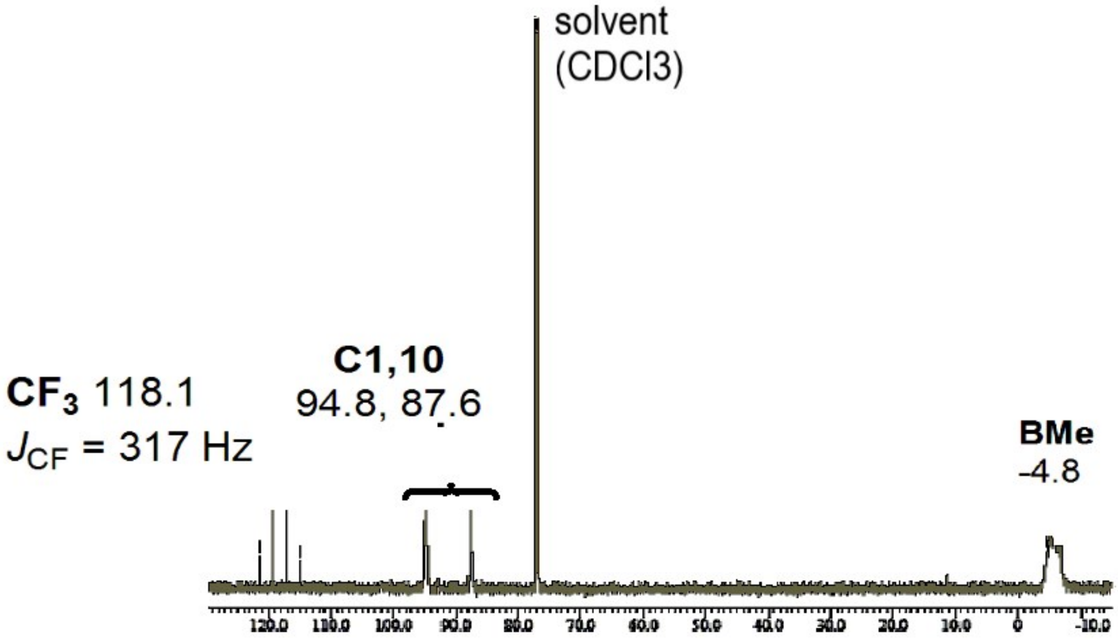

Figure S14. 150.9 MHz ^{13}C-{^{1}H} NMR spectrum of *closo*-1,10-$H_2C_2B_8Me_7$-2-OTf (**5a**). Ordered as assignment/δ(^{13}C in ppm, relative to TMS).

Cartesian coordinates for *closo*-1,10-$H_2C_2B_8Me_8$ at the MP2/TZVP level

	x	*y*	*z*
C	-0.00465	-1.71799	0.59756
B	-0.93171	-1.27693	-0.63233

B	0.93432	-1.27816	-0.62378
B	0.92706	-0.50506	1.07451
B	-0.93903	-0.50378	1.06593
B	0.00656	-0.06157	-1.60627
B	1.32085	0.48427	-0.39931
B	-0.00372	1.03182	0.79549
B	-1.31808	0.48599	-0.41151
C	0.00365	1.31214	-0.78185
H	-0.00732	-2.7028	1.04595
C	-1.95151	-2.28565	-1.30087
C	1.9586	-2.28839	-1.28313
C	-1.96684	-0.66534	2.25839
C	1.94354	-0.66798	2.27646
C	0.01409	-0.07487	-3.18875
C	-2.76222	1.07283	-0.68448
C	2.76806	1.06941	-0.6595
C	-0.00799	2.21661	1.84464
H	0.00641	2.29693	-1.2303
H	-2.52396	0.25416	2.44892
H	-2.69363	-1.45528	2.04715
H	-1.45438	-0.9402	3.18506
H	-2.50378	-1.8275	-2.12382
H	-1.43347	-3.16368	-1.6978
H	-2.68278	-2.64692	-0.57175
H	2.52488	-1.82863	-2.0956
H	2.6774	-2.65781	-0.54578
H	1.44154	-3.16128	-1.69253
H	2.50161	0.25014	2.47078
H	1.42179	-0.93994	3.19879
H	2.66984	-1.46041	2.07302
H	3.55363	0.38093	-0.34165
H	2.92213	1.28004	-1.72193
H	-2.90451	1.29018	-1.74719
H	-3.55132	0.38256	-0.37953
H	-2.91465	2.01056	-0.14195
H	-0.88807	2.85348	1.71524
H	-0.01077	1.85423	2.8746
H	0.87143	2.85541	1.72054
H	2.9146	2.0105	-0.12124
H	0.01253	-1.08964	-3.59174
H	-0.86184	0.44268	-3.59093
H	0.89764	0.43638	-3.58224

Cartesian coordinates for *closo*-1,10-$Me_2C_2B_8Me_8$ at the MP2/TZVP level

C	-1.683432	0.029319	0.005832
B	-0.760123	-0.589950	1.165592
B	-0.731126	1.179253	0.604213
B	-0.748730	0.620092	-1.163514
B	-0.777661	-1.149359	-0.605724

B	0.766468	0.382106	1.245774
B	0.775028	1.237618	-0.402977
B	0.742441	-0.408825	-1.256792
B	0.733620	-1.264508	0.388274
C	1.683479	-0.028823	-0.007648
C	-3.186515	0.042219	0.019538
C	-1.422330	-1.246644	2.443946
C	-1.355353	2.468153	1.276985
C	-1.459779	-2.418138	-1.260864
C	-1.392658	1.293113	-2.442680
C	1.435961	0.807001	2.615158
C	1.361939	-2.648984	0.826509
C	1.453634	2.601037	-0.832989
C	1.378542	-0.853410	-2.635743
C	3.186554	-0.046661	-0.001020
H	-3.561156	0.744023	0.767446
H	-3.582614	0.338098	-0.954033
H	-3.575928	-0.949394	0.258510
H	-0.727549	-3.112590	-1.678082
H	-2.051748	-2.969011	-0.522662
H	-2.142006	-2.130357	-2.066921
H	-0.678143	-1.591996	3.164651
H	-2.078607	-0.537653	2.958501
H	-2.037876	-2.108796	2.167407
H	-0.589450	3.178373	1.595580
H	-2.026571	2.990700	0.588280
H	-1.944945	2.203008	2.160631
H	-0.636263	1.670921	-3.133809
H	-2.013096	0.578452	-2.993029
H	-2.038028	2.133019	-2.166805
H	2.068634	1.691021	2.485637
H	0.695752	1.041614	3.382982
H	2.077002	0.010164	3.005538
H	2.135538	2.456915	-1.676875
H	0.719053	3.354506	-1.124859
H	2.044844	3.019672	-0.011961
H	3.578788	-0.273779	-0.994435
H	3.580625	0.923799	0.307190
H	3.560446	-0.801468	0.693721
H	1.949789	-2.539546	1.743725
H	0.598702	-3.406782	1.015532
H	2.036107	-3.040784	0.058489
H	2.010655	-1.738616	-2.513152
H	0.617705	-1.092467	-3.381734
H	2.010499	-0.062265	-3.051834

Computed ^{11}B NMR (GIAO-MP2/II//MP2/TZVP), in ppm with respect to $BF_3.OEt_2$

-12.6 ppm **4a**
-0.8 ppm **4c**
-2.4 ppm **4d**
